# Evaluating Detection Limits of Next-Generation Sequencing for the Surveillance and Monitoring of International Marine Pests

**DOI:** 10.1371/journal.pone.0073935

**Published:** 2013-09-04

**Authors:** Xavier Pochon, Nathan J. Bott, Kirsty F. Smith, Susanna A. Wood

**Affiliations:** 1 Environmental Technologies, Cawthron Institute, Nelson, New Zealand; 2 Aquatic Sciences, South Australian Research and Development Institute, Adelaide, Australia; 3 School of Biological Sciences, University of Waikato, Hamilton, New Zealand; Institution and Department: Agricultural Research Service, United States of America

## Abstract

Most surveillance programmes for marine invasive species (MIS) require considerable taxonomic expertise, are laborious, and are unable to identify species at larval or juvenile stages. Therefore, marine pests may go undetected at the initial stages of incursions when population densities are low. In this study, we evaluated the ability of the benchtop GS Junior™ 454 pyrosequencing system to detect the presence of MIS in complex sample matrices. An initial *in-silico* evaluation of the mitochondrial cytochrome *c* oxidase subunit I (*COI*) and the nuclear small subunit ribosomal DNA (*SSU*) genes, found that multiple primer sets (targeting a ca. 400 base pair region) would be required to obtain species level identification within the *COI* gene. In contrast a single universal primer set was designed to target the V1–V3 region of *SSU*, allowing simultaneous PCR amplification of a wide taxonomic range of MIS. To evaluate the limits of detection of this method, artificial contrived communities (10 species from 5 taxonomic groups) were created using varying concentrations of known DNA samples and PCR products. Environmental samples (water and sediment) spiked with one or five 160 hr old *Asterias amurensis* larvae were also examined. Pyrosequencing was able to recover DNA/PCR products of individual species present at greater than 0.64% abundance from all tested contrived communities. Additionally, single *A. amurensis* larvae were detected from both water and sediment samples despite the co-occurrence of a large array of environmental eukaryotes, indicating an equivalent sensitivity to quantitative PCR. NGS technology has tremendous potential for the early detection of marine invasive species worldwide.

## Introduction

The introduction of marine invasive species (MIS) can dramatically modify indigenous biodiversity and habitats [1]–[4]. The altered community may undergo degradation of associated ecological, economic and social values [4], [5]. As a consequence, the prevention of ecological invasions has become a priority for many governments, especially in island nations [6]–[7]. For example, New Zealand has a targeted surveillance programme for six marine pests and these are currently listed on the register of Unwanted Organisms under the Biosecurity Act 1993, including the European shore crab *Carcinus maenas*, Chinese mitten crab *Eriocheir sinensis*, Northern Pacific seastar *Asterias amurensis*, Mediterranean fanworm *Sabella spallanzanii*, Asian clam *Corbula amurensis*, and the marine aquarium weed *Caulerpa taxifolia*. Of these species, *S. spallanzanii* is already present in New Zealand [8] and *C. taxifolia* has been identified from aquariums on multiple occasions [9]. Surveys are conducted twice yearly at 12 high-risk locations throughout New Zealand using SCUBA-diving transects, shore searches, crab condos, starfish traps and benthic sleds [10]. Collected specimens are then morphologically identified to species or the lowest possible taxonomic unit.

The detection of MIS soon after an incursion, when populations are still confined to a small area and at a low density, maximizes the probability of effective management [11]. Early detection of MIS has been problematic because morphological identification of some life-history stages, especially larvae, is challenging and requires very specific taxonomic expertise [12]. Consequently, there are few surveillance programmes that monitor the water column for dispersive life forms (e.g., planktonic larvae) of MIS. Molecular techniques have the potential to be faster, more specific, and have greater standardization. Molecular methods also reduce the problem of a growing shortage of specialist taxonomists [13].

The development of molecular based methods that target dispersive life stages of marine organisms in the water column is now seen as an effective strategy for detecting MIS in surveillance [14], [15]. In recent years, a number of molecular methods have been developed for targeting high profile MIS at the larval stage, including *Fluorescent* In Situ *Hybridization* (FISH; [16], [17]), *Sandwich Hybridization Assays* (SHA; [18]–[21]), *PCR-based DNA fingerprinting* [22]–[24], and *Quantitative PCR* (qPCR; [25]–[27]). Despite the great potential of these tools [15], a number of restraints have also been identified, including the relatively limited scope for analyzing samples in multiplex and the reduced sensitivity for detecting low abundance targets such as planktonic larvae (e.g., SHA).

**Figure 1:**
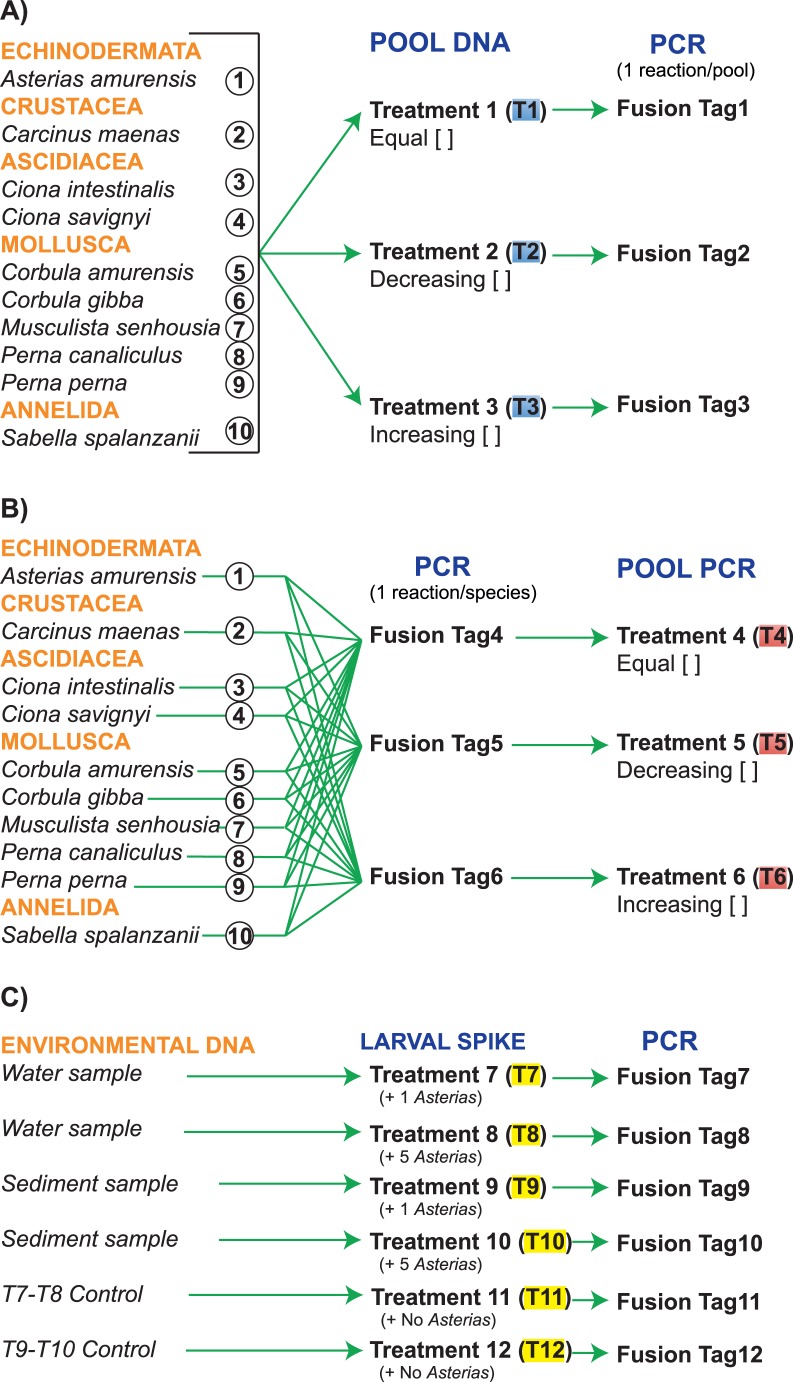
Detailed experimental design. **A**) DNA samples from ten species were pooled together at equimolar (T1), decreasing (T2), and increasing concentrations (T3); each treatment was then PCR-amplified using specific fusion primers. **B**) Three distinct PCR-amplifications were run for each species individually, using distinct fusion primers. PCR products with identical primer tags were then pooled together at varying concentrations (T4, T5, T6). **C**) One water and one sediment samples, were collected; each sample was divided into three sub-samples and spiked with either 1 larva (T7, T9), 5 larvae (T8, T10), or no larva (controls T11, T12) of the Northern-Pacific seastar *Asterias amurensis* (genus *Asterias* does not occur in New Zealand), and PCR-amplified. Treatments 1 to 6 and 7 to 12 were pooled together and analysed in multiplex on the 454 GS Junior™ pyrosequencer.

Next-Generation Sequencing (NGS) has drastically modified scientific approaches in basic, applied and clinical research [28]–[30]. It provides the potential for a step-change approach to environmental surveillance methods through its ability to identify a wider range of taxonomic groupings [31]. The major advance offered by NGS is the ability to produce enormous volumes of sequence (DNA or RNA) data cheaply. Despite the increasing use of NGS for monitoring biodiversity from environmental samples such as soil, water, sediment, faeces, or air [32]–[36], very little is known about the detection limits afforded by NGS technologies as well their accuracy for estimating the relative proportions of organisms present at different abundances within a sample. In this study, we assessed the detection limit of the Roche 454 GS Junior™ pyrosequencing system for use in marine biosecurity monitoring by creating artificial communities of ten MIS using varying concentrations of known DNA samples and PCR products, as well as environmental samples (water and sediment) spiked with one or five larvae of *A. amurensis* (Figure 1). A ca. 400 base-pair (bp) region of the 18S nuclear small subunit (*SSU*) ribosomal DNA gene was analyzed in multiplex using barcodes.

## Results

### Sanger Sequencing and Marker Selection

Four distinct primer sets were required to obtain positive PCR amplifications for the *COI* gene across the five taxonomic groups using the same thermocycling conditions. High-quality sequences were only obtained for seven of the twelve species (Table 1). Examination of the approximately 600 bp *COI* sequence alignment containing 221 sequences revealed high sequence variability among species (data not shown; alignment available in DNA S1), and demonstrated that at least five distinct primer sets (including degenerated primers) would be required to obtain an approximately 400 bp fragment of this gene. No further experiments were undertaken using the *COI* gene.

**Table 1:** The twelve Marine Invasive Species (MIS) investigated in this study.

Species^a^	Taxonomic Group	Sample type	Collection localities	Collection date	*COI*	*SSU*
*Asterias amurensis* (1)	Echinodermata	Gonads	Hobart, Australia	May-09	HG005368	HG005356
*Carcinus maenas* (2)	Crustacea	Leg tissue	Mullaghmore, Ireland	April-11	N/A	HG005357
*Ciona intestinalis* (3)	Ascidians	Stomach muscle	Nelson, New Zealand	Aug-10	N/A	HG005358
*Ciona savignyi* (4)		Gonads	Nelson, New Zealand	Aug-10	N/A	HG005359
*Didemnum vexillum*		Tissue section	Lyttelton, New Zealand	Mar-09	N/A	HG005366
*Eudistoma elongatum*		Tissue section	Rangaunu harbor, New Zealand	February-09	HG005369	HG005367
*Corbula amurensis* (5)	Mollusca	Tissue section	San Francisco, USA	August-09	HG005370	HG005360
*Corbula gibba* (6)		Tissue section	Bay of Morlaix, France	March-09	HG005371	HG005361
*Musculista senhousia* (7)		Tissue section	Auckland, New Zealand	September-10	HG005372	HG005362
*Perna canaliculus* (8)		Tissue section	Nelson, New Zealand	September-10	HG005373	HG005363
*Perna perna* (9)		Tissue section	Tasman Bay, New Zealand	March-08	HG005374	HG005364
*Sabella spalanzanii* (10)	Annelida	Tentacle	SARDI, Australia	October-09	N/A	HG005365

List indicates the taxonomic group of each MIS and corresponding sample type, collection localities and date, and the GenBank accession numbers of *COI* and *SSU* sequences.

a Numbers in brackets correspond to the species reference number shown in Figure 1.

Analysis of sequence alignments of the *SSU* gene (DNA S2) allowed a single set of primers (18S_1F and 18S_400R) to be designed that enabled amplification of an approximately 400 bp fragment, including the V1–V3 hypervariable regions. Using these primers, amplicons of correct size and sequences were obtained for all twelve species (Table 1). An analysis of the phylogenetic resolution of this *SSU* fragment demonstrated that the uncorrected genetic divergence between species was relatively low and ranged between 0.003 and 0.336 (Table S1) compared to between 0.144 and 0.479 for the *COI* gene (data not shown). The lowest value (0.003) corresponded to a single bp change found between the *Perna canaliculus* and *P. perna* sequences. GenBank accession numbers for the obtained Sanger sequences of *COI* and *SSU* are indicated in Table 1.

### 454 Analysis of Contrived Communities (Treatments 1 through 6)

Out of the twelve marine invasive species listed in Table 1, two species (*Didemnum vexillum* and *Eudistoma elongatum*) failed to PCR-amplify using the primers 18S_1F and 18S_400R with fusion tags despite successful amplifications using the standard primers. After multiple trials, these two species were excluded from further analysis, and only ten species were included in the contrived community experiments (see Figure 1A–B).

Pyrosequencing of treatments 1 through 6 generated 53,576 sequences after quality filtering, size trimming, separation by barcodes, and chimera filtering. Between 5,089 (treatment 2) and 12,931 (treatment 5) sequences per treatment were obtained (mean = 8,929+/−2,370). The 454 pyrosequencing reads for treatments 1 to 6 are available in DNA S3.

The local BLASTn search of treatments 1 through 6 separated all investigated species except for the two *Perna* species, whose sequences were indistinguishable regardless of the e-value threshold employed. The e-value is a parameter that describes the number of “expected” hits that occur by chance when searching a database of a particular size. A total of 92.4% of sequence reads (N = 53,268) resulted in a BLASTn e-value of zero while the remaining reads (N = 308; 7.6%) generated e-values greater than 10^−107^. Detailed examination of all *Perna* spp. sequences recovered from treatments 1 through 6 (N = 15,456) revealed that 77.6% of sequences (N = 11,988) were an exact match to the *P. canaliculus* Sanger sequence (Table 1; GenBank HG005363) while only 0.03% of sequences (N = 5) matched the *P. perna* Sanger sequence (Table 1; GenBank HG005364). The remaining 22.4% of sequences (N = 3463) differed from the *P. canaliculus* sequence by 1 to 15 bp changes, none of which corresponded to the single mutation site originally separating the *P. canaliculus* and *P. perna* sequences. Consequently, all *Perna* spp. sequences within each treatment were grouped together and enumerated as indicated below.

**Figure 2:**
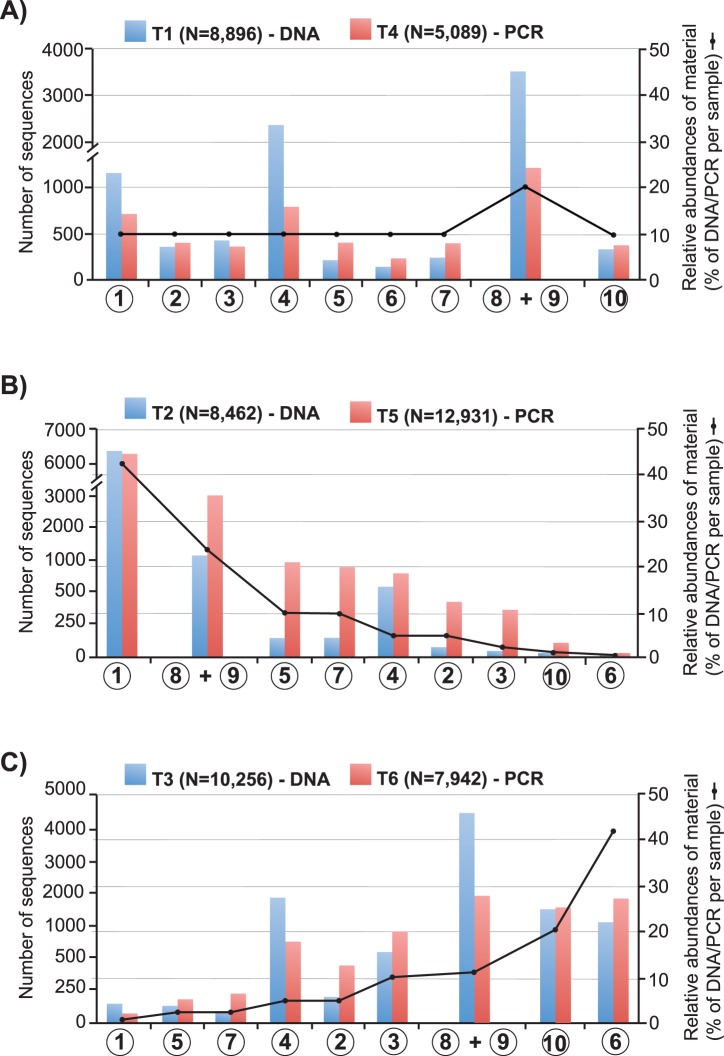
Contrived community experiment. Histograms show the number of recovered sequences per investigated species (circled numbers; see Figure 1) and for pooled DNA (T1, T2, T3) and pooled PCR (T4, T5, T6) treatments at **A**) equimolar, **B**) decreasing, and **C**) increasing concentration of starting material (shown in relative abundance of DNA/PCR products; see doted line and vertical scale on right of graphs). Two species, *Perna perna* and *Perna canaliculus*, were pooled together due to lack of *SSU* marker differentiation.

Between 140 and 3,498 and between 225 and 1,404 sequences per species were recovered from the equimolar concentration of treatments T1 and T4, respectively (Figure 2A). Marked differences in sequence number per species were observed, in particular the comparatively high number of sequences obtained from species 1, 4, and 8 (*A. amurensis*, *C. savignyi*, and *Perna* spp.) which collectively represented 80.5% and 57.0% of sequences obtained from all species in the pooled DNA (T1) and pooled PCR (T4) treatments, respectively. Between 7 and 6,435 and between 18 and 6,345 sequences per species were recovered from the varying concentration DNA (T2, T3) and PCR (T5, T6) treatments, respectively (Figure 2B, 2C; Table S2). There were strong correlations between the number of sequences obtained and the relative abundance of starting DNA/PCR material in the decreasing concentration treatments T2 (F_1,11_ = 35, *p* = 0.0005, *r^2^* = 0.833) and T5 (F_1,11_ = 369, *p* = <0.0001, *r^2^* = 0.981) (Figure S1A, S1C). In comparison, significant variability was observed in the number of sequences obtained in the increasing concentration treatments T3 (F_1,11_ = 0.42, *p* = 0.539, *r^2^* = 0.056) and T6 (F_1,11_ = 11.8, *p* = 0.01, *r^2^* = 0.627) (Figure S1B, S1D). Species 1, 4, and 8 also showed higher than expected number of sequences compared to other species, especially within the pooled DNA treatments (T2, T3; Figure 2B, 2C). Within treatment T3, for example, species 4 (*C. savignyi*) represented only 5.2% abundance of the DNA pool mixture but actually generated a number of sequences most similar to species 10 (*S. spalanzanii*) and which represented 20.7% of the DNA pool mixture (see Table 2A, Figure 2C). The increasing/decreasing contrived community experiments showed that both species 6 (*C. gibba*; treatments T2 and T5) and species 1 (*A. amurensis*; treatments T3 and T6) were detected despite representing ca. 0.6% of the pooled DNA/PCR mixtures.

**Table 2:** Relative abundances of genomic DNA/PCR products used in Treatments 1–6.

Species^a^	Equimolar - Treatments T1 &T4	Decreasing - Treatments T2 & T5	Increasing - Treatments T3 & T6
*Asterias amurensis* (1)	10	41.29	0.64
*Carcinus maenas* (2)	10	5.16	5.16
*Ciona intestinalis* (3)	10	2.58	10.32
*Ciona savignyi* (4)	10	5.16	5.16
*Corbula amurensis* (5)	10	10.32	2.58
*Corbula gibba* (6)	10	0.64	41.29
*Musculista senhousia* (7)	10	10.32	2.58
*Perna canaliculus* (8)	10	20.65	1.29
*Perna perna* (9)	10	2.58	10.32
*Sabella spalanzanii* (10)	10	1.29	20.65

Table indicates the relative abundances (percent per sample) of genomic DNA and PCR products used in Treatments 1 through 6 (see Figure 1A–B).

a Numbers in brackets correspond to the species reference number shown in Figure 1.

### Quantitative PCR and 454 Analyses of Environmental Samples (Treatments 7 through 12)

The results of qPCR analysis of treatments T7, T8, T10 and T11 were positive and confirmed that *A. amurensis* larva had been spiked into these samples. No *A. amurensis* signal was obtained in control samples T9 and T12.

Pyrosequencing of treatments 7 through 12 generated 70,796 sequences after filtering. Between 9,977 (treatment 9) and 13,908 (treatment 12) sequences per treatment were obtained (mean = 12,169+/−1,482). The 454 pyrosequencing reads for treatments 7 to 12 are available in the DNA S4.

**Figure 3:**
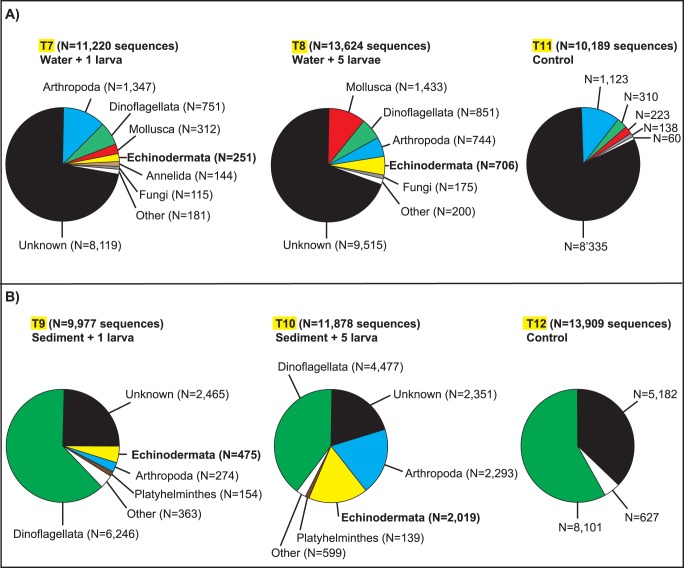
Environmental DNA/Spiking experiment. Pie charts depict the proportion of marine phyla identified via BLASTn searches from environmental samples spiked with 1 larva (T7, T9), 5 larvae (T8, T10) or no larva (controls T11, T12) of *Asterias amurensis* (i.e., phylum Echinodermata; shown in bold) from **A**) one water and **B**) one sediment sample. A detailed taxonomic list of marine taxa, sequences lengths, minimum e-values, mean similarity, and number of best sequence hits to known NCBI sequences are shown for each treatment in Table S2.

The environmental water sample spiked with one *A. amurensis* larva (T7) produced 11,220 sequence reads (Figure 3A), representing 74 distinct species classified in over 15 distinct phyla (Table S2A). Approximately 72% of sequences (N = 8,119) corresponded to uncultured/unknown organisms. The water sample spiked with five *A. amurensis* larvae (T8) produced 13,624 sequence reads, representing 76 distinct species in 14 distinct phyla, of which approximately 70% (N = 9,515 sequences) matched uncultured/unknown organisms (Figure 3A, Table S2B). The proportion of the most commonly identified phyla varied between the three water samples (Figure 3A). Arthropoda, Dinoflagellata, and Mollusca were most common in treatment T7 (N = 1,347, N = 751, and N = 312 sequences) and in the control treatment T11 (N = 1,123, N = 310, and N = 223 sequences), respectively. In treatment T8 the most common encountered phyla were Mollusca (N = 1,433), Dinoflagellata (N = 851), and Arthropoda (N = 744). All sequences matching Echinodermata species corresponded to one of three Asteriidae species (*A. forbesii*, *Diplasterias brucei*, and/or *Pisaster ochraceus*) (see Table S2). A careful examination of sequences showed that GenBank sequences for *D. brucei* (DQ060785) and *P. ochraceus* (DQ060813) were one and two bp different from the generated Echinodermata sequences (Table 1), an observation that was also reflected in the slightly lower minimum e-values obtained for these species compared to *A. forbesii* (see Table S2). The latter sequence on the other hand resulted in exact matches to our reference sequence of *A. amurensis*. Only two complete SSU sequences of *A. amurensis* (D14358 and DQ206636) are available in GenBank but both are trimmed on the 5′-end such that there are at least 40 bp that do not overlap with the query sequences, hence this explains why generated Echinodermata sequences resulted in closer BLASTn hits with *A. forbesii* and not *A. amurensis*. No Echinodermata sequences were recorded in the control treatment T11. For the reasons mentioned above, and because none of these species (*A. forbesii*, *D. brucei*, and *P. ochraceus*) occur in New Zealand [37], we have combined all Asterididae hits and considered them as representing the *A. amurensis* larvae that were spiked into the samples, hereafter are referred to as *Asterias* sp. Treatments T7 and T8 contained 251 (2.24%) and 706 (5.18%) sequences of *Asterias* sp., respectively.

The sediment sample spiked with one *A. amurensis* larva (T9) generated 9,977 sequence reads (Figure 3B), representing 91 distinct species classified in over 19 phyla (Table S2D). Approximately 25% of sequences (N = 2,465) corresponded to uncultured/unknown organisms. The sediment sample spiked with five *A. amurensis* larvae (T10) generated 11,878 sequence reads, representing 84 distinct species in 21 distinct phyla, of which approximately 18% (N = 2,351 sequences) matched uncultured/unknown organisms (Figure 3B, Table S2E). Treatments T9 and T10 contained 274 (2.74%) and 2,019 (17%) sequences of *Asterias* sp., respectively. The proportion of the most commonly identified phyla varied between the three sediment samples (Figure 3B). Dinoflagellata largely dominated all samples with 6,246, 4,077, and 8,101 sequences detected in treatments T9, T10, and T12, respectively. The presence of Arthropoda and Platyhelminthes was only evident in treatments T9 and T10, but below the 1% threshold of the pie chart in the control treatment T12 (i.e., white portion of the pie charts in Figure 3). Overall diversity differed between water and sediment habitats. The diversity of dinoflagellate species detected in the sediment was approximately twice that recorded in the water column (see Table S2). Furthermore, differential diversity of dinoflagellate genera was also observed, with *Ceratium*, *Dinophysis*, *Protopteridinium* and *Thecadinium* dominating in the water column, and *Alexandrium*, *Gonyaulax*, and *Scrippsiella* dominating in the sediment.

## Discussion

### NGS Platforms and Marker Choice: Promises and Pitfalls for Routine Monitoring of Marine Invasive Species

DNA metasystematics, *i.e*. the identification of massive amounts of barcode DNA sequences obtained via NGS technology from environmentally derived samples, is becoming the tool of choice for understanding the evolutionary history of interacting species and ecological biodiversity in environmental samples [35], [38]. NGS technologies have facilitated analysis of community structure and biodiversity assessments of a range of eukaryotic and prokaryotic assemblages from marine, freshwater, and terrestrial ecosystems [32], [33], [39]–[42], providing unprecedented and valuable information on environmental health and biodiversity. Despite the tremendous potential of NGS technology for the monitoring of high-risk bioinvading organisms, such approach has never been specifically investigated for MIS. To our knowledge the sensitivity of 454 pyrosequencing for detecting eukaryotic species present in low abundance from aquatic communities has only been addressed twice [32], [36]. In this study, we tested the ability of the GS Junior™ 454 pyrosequencing system to detect low abundance MIS from a set of artificially constrained communities and environmental samples spiked with a known number of larvae. The overarching goal was to establish an effective and financially viable diagnostic tool to be incorporated into routine marine biosecurity monitoring programs.

Our initial efforts focused on identifying a suitable barcoding marker with the capacity to differentiate all investigated species using universal primers, and to reduce analysis cost by designing a multiplex approach using the GS Junior™ 454 platform. Compared to other 454 platforms, the GS Junior™ is a user-friendly benchtop and the most affordable pyrosequencing system in terms of instrument and reagent costs (∼US$1500 per run) while still being able to generate ∼0.1 millions of sequence reads per run in less than 10 hours [43]. The main limitation of the GS Junior™ currently lies in its average read length capacity of ca. 400 bp, which dictates the genetic markers and target primers that can be used.

To be used as a DNA metabarcode, a genome locus should be flanked by highly conserved regions to allow design of universal primers that can amplify DNA from the target organism group(s) with minimal bias [44]. They must also contain variable regions that allow discrimination over a wide range of taxonomic levels. The preferred approach is to target a barcoding gene that is informative at the species level and for which a large diversity of organisms have been sequenced and documented. We investigated the use of both mitochondrial *COI* and nuclear *SSU* genes because sequence data of these genes are amongst the most voluminous components of public databases [45], [46]. These regions are increasingly being used for barcoding a wide diversity of life forms ranging from Archaea/Eubacteria to Eukaryotes [47]–[50].

The *COI* gene is one of the most popular markers for population genetics and phylogeographic studies across the animal kingdom [51], [52], and a ca. 650 bp fragment known as the Folmer region [53] has been shown to be an efficient species-level identification tool for a variety of metazoan species from terrestrial, marine and freshwater environments [54]–[56]. The high abundance of mitochondrial DNA (mtDNA) copies per cell [57] also potentially allows rare species to be more easily detected. However, *COI*-based DNA barcoding sometimes faces problems, including poor species resolution in some taxa within Porifera, Anthozoa, fungi and plants [58]–[60], and/or poor PCR amplification in some groups such as, for example, nematodes [61]–[63]. In this study, the Folmer primers failed to amplify all investigated marine invasive species and three additional *COI* primer sets were required to obtain positive amplifications for Echinodermata, Crustacean, and Ascidiacea species. Several sequences, however, were unreliable due to poor direct sequencing quality suggesting a lack of primers specificity (Table 1), and the design of a universal primer set targeting an ca. 400 bp fragment of the Folmer region was impossible due to excessive sequence variability among investigated species. Recent research involving mitochondrial isolation by enrichment [64] coupled with the forecasted capacity of Roche 454 GS FLX+ systems to produce high-quality sequence reads of more than 1000 bp (http://454.com/seewhatspossible/) are promising avenues for developing future metasystematics approaches using mitochondrial genes.

In contrast, the *SSU* gene yielded high-quality direct sequences for all investigated species (Table 1), and the succession of conserved and hypervariable DNA regions along this ribosomal gene provided ample opportunities for the design of universal primer sets. Previous metasystematics studies using 454 pyrosequencing for assessing eukaryotic diversity from aquatic ecosystems have targeted different SSU regions. For example, Chariton et al. [31] targeted ca. 200–500 bp fragments located at the 3′-end of SSU to explore meio and macro fractions of estuarine sediments, while Zhan et al. [36] focused on ca. 400–600 bp fragments encompassing the hypervariable V4 region of *SSU* (V4-*SSU*) to detect rare species in the water column. Despite appreciable level of biodiversity recovered in these previous studies using universal primers, both exceeded 400 bp, hence were not applicable in our study. Despite the ability of our universal primer set (V1–V3-*SSU*) to amplify a wide range of taxonomic groups, including animals, plants (algae), fungi and protists (Figure 3; Table S2), the species-level resolution was poor in some groups. For example, this marker was able to successfully differentiate the two *Ciona* and *Corbula* species studied here with genetic divergences of 1.4% and 16.8% (Table S1), respectively, but could not discriminate between the invasive South African brown mussel (*P. perna*) and the New Zealand green-lipped mussel (*P. canaliculus*). Examination of other SSU regions, including the V4-*SSU* used by Zhan et al. [36], also yield identical DNA sequences for both *Perna* species (data not shown), indicating that *SSU* gene is inappropriate for species-level differentiation within this genus and highlighting the need to adapt this NGS tool to more variable markers. Considering the relatively low interspecific variability reported here combined with the estimated percent of errors per base using 454 pyrosequencing (∼1% [43]), further *in silico* analysis will be required to test the likelihood of mistaking an invasive species with local-related fauna, especially when interpreting community assemblages based on the generation of Operational Taxonomic Units (OTU) through prior clustering steps of sequence data [e.g., 31, 36].

A major advantage of NGS metasystematics is the capability for simultaneous detection of a variety of target taxa based on actual DNA sequences, reducing the potential for type I errors (*i.e*., a positive assay result but the target species is not present). These errors can be very problematic using PCR-based diagnostic tools such as gel-based DNA fingerprinting or qPCR [65]. The use of NGS, however, does not necessarily eliminate type II errors (i.e., failure to detect the target species when it is present). A unexpected example of a type II error encountered in this study was the successful PCR amplification of *D. vexillum* and *E. elongatum* using the standard SSU primers 18S_1F and 18S_400R but the failure to amplify these ascidian species using the same primer set with fusion tags. Berry et al. [66] showed that adaptors and barcode nucleotide sequences adjacent to the template-specific PCR primers can sometimes interact with the template strand in such a way as to promote template-sequence dependent selective amplification. Additional research is underway with the aim to minimize the potential for type II errors, including the testing of fusion primers that detect all key MIS using various template dilutions and thermo-cycling conditions.

### High Sensitivity of 454 GS Junior™ System for the Detection of Low Abundance Marine Pests

Results from our artificially constraint species communities showed that all DNA/PCR samples present at greater than 0.64% abundance of the pooled mixtures could be detected. This contrasts with Hajibabaei et al. [32] who reported a 1% detection threshold using pyrosequencing on freshwater benthic macroinvertebrate taxa. It is possible that the increased sensitivity observed here is due to the use of a relatively low number of interacting species compared to Hajibabaei et al. [32] who pooled 255 specimens representing 23 distinct species of Ephemeroptera and Trichoptera. Although additional contrived community experiments are needed to test detection limit variations over a wider range of interacting species, our preliminary data are very promising and indicate that pyrosequencing is a powerful tool for the detection of low abundance taxa from mixed communities. Another consideration relates to quantitative accuracy of pyrosequencing. Despite incorporating many of the techniques known to reduce PCR biases, including high template concentration (>4 ng/µl), low cycle numbers (<30), and the use of high-fidelity DNA polymerase (e.g., [67]), our results showed considerable variability in the number of sequence reads obtained between species and between sample types (i.e., DNA versus PCR treatments). For example, *A. amurensis*, *C. savignyi*, and *Perna* sp. consistently yielded higher number of sequences in all treatments, especially within DNA treatments, and this observation was particularly striking at equimolar concentration (Figure 2A). The most likely explanation for these observed differences is that the three species contain higher *SSU* copy numbers, which enhanced their amplification over other species. Weber and Pawlowski [68] recently demonstrated that the rDNA copy number and rRNA expression level in foraminifera have a strong impact on the proportion of sequences derived from rDNA and cDNA libraries, and that it was impossible to accurately determine abundance of species based on *SSU* sequence abundance without prior assessment of the copy number per individual and normalization. Since this method would be impractical for environmental monitoring, NGS metasystematics has limited scope for accurate quantitation of species but is powerful for assessing the biological richness of a sample [69] and is well suited for most surveillance programmes which rely on detection of unwanted/invasive species rather than quantification. A further explanation is that PCR artifacts such as differential amplification efficiency [67], [70], [71] are responsible for the observed biases. The fact that both DNA and PCR treatments of *A. amurensis*, *C. savignyi*, and *Perna* spp. generally yielded higher sequence abundance compared to other species at similar concentration reinforces this second scenario and similar observation have been made by other researchers [e.g., 72].

Results from the spiking experiment of environmental samples showed that a single *A. amurensis* larva could be detected from both water and sediment samples (Figure 3), indicating that GS Junior™ pyrosequencing offers similar to or possibly higher sensitivity than qPCR. For example, Smith et al. [27] developed a qPCR assay able to reliably detect one larva of the Asian clam *C. amurensis* in up to 10 g of sediment, but could only detect a minimum of five larvae when inoculated within a more complex environmental matrix consisting of benthic invertebrate and macro-algal assemblages. Zhan et al. [36] also investigated the sensitivity of pyrosequencing on artificial gradients of four larval species per freshwater plankton sample, and showed that pyrosequencing could reliably detect >1 larva per sample, but their results were inconsistent at lower concentration suggesting that detection limit was reached at around 1 larva per sample. In concert, these studies suggest that pyrosequencing is a powerful technology for the detection of low abundance and early life stages MIS, which are difficult/impossible to identify morphologically.

## Conclusion

Current biosecurity surveillance programs are not designed to provide an effective monitoring strategy for the early detection of MIS at larval or juvenile life stages, particularly in the water column [15]. Developing the capacity to accurately and rapidly detect new incursions of marine pests using environmental NGS may allow appropriate remedial actions to eradicate these incursions before they spread. In this study, we demonstrated that the 454 GS Junior™ pyrosequencing system can effectively detect low abundance species (i.e., 0.64% of pooled mixtures) from artificially constrained MIS communities, as well as is able to recover sequences from single *A. amurensis* larvae artificially spiked into planktonic and benthic environmental samples. Despite a number of challenges also identified in this study, including poor resolution of the *SSU* marker for some taxa and potential PCR-based biases complicating interpretation of actual relative abundance in mixed communities, the high sensitivity reported here has tremendous potential for the early detection of low abundance MIS. We predict that the anticipated rapid decrease in price and concurrent improvement in quality, precision and fragment sizes of NGS technologies will soon allow the transfer of our multiplex approach to more informative markers (e.g., *COI*). NGS will improve biosecurity monitoring programmes by (*i*) allowing for the detection of multiple invasive species simultaneously, (*ii*) providing a sensitive presence-absence tool for the detection of a wide range of marine organisms and (*iii*) visualizing ecological and distributional changes within biotic communities. This will enable the rapid identification of novel biosecurity threats and increase the likelihood of the successful eradication of new incursions.

## Materials and Methods

### Marker Evaluation, DNA Extraction, PCR, and Sequencing

Two distinct barcoding genes were investigated for twelve marine species (Table 1): an approximately 600 bp portion of the mitochondrial *cytochrome* c *oxidase I* (*COI*), and an approximately 600 bp portion of the nuclear *SSU* (including variable regions V1–V3). For each marker, a ‘global’ sequence alignment database was generated for the design of PCR primers by grouping representative *COI* (<900 bp) and *SSU* (<2500 bp) sequences of each species (and closely related species) from GenBank (National Center for Biotechnology Information, NCBI). Sequences were aligned using ClustalW [73] in BioEdit Sequence Alignment Editor [74] and further editing undertaken by manual inspection.

Specimens (Table 1) were collected from various locations, and stored at 4°C in 95% EtOH until processing. Specimens from outside of New Zealand were collected under permits of corresponding authorities (see Acknowledgments), and were imported into New Zealand under MAF permit for importation of non-viable specimens (#2011043308). No specific permissions were required either for the specimens collected in New Zealand or for the samples of *Carcinus maenas* and *Sabella spalanzanii*. None of the collected marine invasive species involved endangered or protected species.

Total genomic DNA was extracted for two specimens of each species using the PureLink™ Genomic DNA Mini Kit (tissue protocol; Invitrogen). PCR-amplification trials were conducted for each species and marker. The *COI* gene was PCR-amplified using the *LCO1490* and *HCO2198* primers and thermocycling conditions described in Folmer et al. [53]. This primer set failed to amplify all species and a series of other species or group specific primers were used. These included the *TUN_F* and *TUN_R* primers [75] for ascidians, the *CRUS_F1* (5′-TCTACAAATCATAAAGAYATTGGHAC-3′) and *CRUS_R1* (5′-CYATHCCNACHGKAAATATRTGRTGRGC-3′) designed in this study for crustaceans, and the *ASTER_F1* 5′-(GCTGGTATGATTGGAACTGCT-3′) and *ASTER_ R1* (5′-AACAGTAAACATATGGTGAGC-3′) designed in this study for the Northern-Pacific seastar, *A. amurensis*. The *SSU* was PCR-amplified from all specimens using the following primers designed in this study *18S_1F* 5′- GCCAGTAGTCATATGCTTGTCT-3′ and *18S_701R* 5′- GGAGCTGGAATTACCGC-3′.

PCR amplifications were carried out in 50 µl reaction volumes containing 25 µl of i-Taq 2× PCR master mix (Intron, Gyeonggi-do, Korea), 0.4 µM of both primers and approximately 20–180 ng of template DNA. A touchdown PCR protocol was performed on a BioRad iCycler™ using the following conditions: 3 min at 95°C, 20 cycles of 94°C for 30 s, 62°C for 30 s (decreased by 0.5°C at each cycle), 72°C for 1 min, and followed by 12 additional cycles with an annealing temperature set at 52°C, and a final extension of 72°C for 7 min. Amplification products were purified using AxyPrep PCR cleanup kits (Axygen, California, United States) and sequenced bidirectionally by an external contractor (Genetic Analysis Services, University of Otago, New Zealand) using an ABI 3100 Genetic Analyzer (Applied Biosystems, California, United States). Bi-directional sequence chromatograms were inspected and assembled using Geneious v5.5.6 (Biomatters Ltd, New Zealand) and compared to other sequences in NCBI using the Basic Local Alignment Search Tool (BLASTn).

The aligned sequences were evaluated for conserved gene regions that would enable the design of a single primer pair while amplifying all target species, but not exceeding the average read length capacity of the 454 GS Junior™ instrument (i.e., ca. 400 bp [43], while allowing sufficient phylogenetic resolution for species differentiation. Within the *COI* it was not possible to design primers that met these selection criteria (see results) and this gene was not used for further experiments. The *SSU* sequences shared a conserved region located approximately 400 bp downstream of the *18S-F1* primer, and an internal reverse primer *18S_400R* (5′-GCCTGCTGCCTTCCTT-3′) was designed. Primers *18S_1F* and *18S_400R* overlap with the previously employed 18S primers SSU_FO4 and SSU_R22, respectively 7678. All DNA samples were tested with primers *18S_1F* and *18S_400R* using the touchdown PCR protocol described above. Estimates of uncorrected genetic divergence between species based on the approximately 400 bp *SSU* fragment were calculated using the program MEGA v4.0 [79], using the p-distance model and the pairwise-deletion option.

### Collection of Environmental Samples, DNA Extraction and Quantitative PCR Analysis

A plankton net (20-µm mesh size) sample was collected (May 1, 2012) from a depth of 10 m to the surface in Tasman Bay, New Zealand (S 41.05, E 173.09′). Sub-samples (150 ml) were preserved immediately with 200 ml of RNAlater® (Life Technologies, California, United States) and stored at 4°C until processed. A sediment sample was collected (August 8, 2012) from the same Tasman Bay location from a depth of 11 m using a perspex sediment corer (13 cm diameter), and subsamples of 5 g of sediment were immediately placed in 40 ml of RNAlater® and stored at 4°C until processed.

Three subsamples (50 ml; which equated to 0.23 m^3^ of water concentrated per sample) from the plankton net samples were centrifuged (2500×*g*, 10 min) and the remaining pellets used for further experiments. Excess RNAlater® was decanted from three sediment samples and these were washed twice with Milli-Q water. Either one larva or five *A. amurensis* larvae (120 hr old) of *A. amurensis* originally collected from Hobart, Australia, were stored in saline ethanol fixative [80] and transferred using micro-pipettes and an inverted microscope (CKX41, Olympus, Wellington, New Zealand) to a single tube of PowerMax® Soil DNA isolation kit (Mo Bio, California, United States). Pellets from the water samples or 25 mg of sediment were added to this tube. Control samples in which no larva was added were included for each treatment. DNA was isolated as described in the manufactures protocols.

Quantitative PCR assays were undertaken on all environmental samples to confirm that *A. amurensis* had been successfully spiked into the samples. Analyses were undertaken on a Rotor-Gene 6000 (Corbett, Australia) in 25 µl reactions containing; 12.5 µl of Platinum® Quantitative PCR SuperMix-UDG (Invitrogen, California, United States), 200 nM of forward (Ast_TaqF) and reverse (Ast_TaqR) primers, 160 nM probe (Ast_TaqMGB) (primers and probe from Bax et al. [81], and 2 µl of DNA template. Each sample was analysed in duplicate and no template control and positive control samples were included. Assays were run in clear 0.2 ml thin-wall PCR tubes (Axygen, California, United States). PCR cycling used the following conditions: 50°C for 2 min, 95°C for 2 min and 45 cycles of 95°C for 15 s and 60°C for 45 s.

### Experimental Design of 454 Multiplex, Dilutions, and Fusion Primers

Experiments were designed to test the detection limits afforded by the Roche 454 GS Junior™ pyrosequencing system using the *SSU* gene as a metabarcode [35]. Three sample types were tested; contrived communities using extracted genomic DNA, contrived communities using PCR products and environmental samples spiked with known number of larvae. Due to the lack of PCR amplification of two species (see results), a total of ten out of twelve species listed in Table 1 were included in these experiments (see Figure 1A and 1B). Nucleic acids were quantified both spectrophotometrically using a NanoPhotometer (Implen, Munich, Germany) and fluorometerically using QuBit dsDNA HS Assay (Invitrogen, California, United States). Genomic DNA and PCR product concentrations ranged between 10.5 and 467 ng/µl and between 11.8 and 59.2 ng/µl, respectively. In the first set of treatments (Figure 1A), genomic DNA from the ten marine species were artificially pooled at either equal, increasing or decreasing concentrations (Table 2A), and each treatment was then PCR-amplified using different combinations of forward and reverse 454 HPLC-grade fusion primers with Multiplex Identifier sequence for Differentiation [MID], hereafter referred to as Fusion Tags (Table S3), to allow identification of individual samples in pooled sequencing runs. All primers were adapted with either the “Primer A” (forward) or “Primer B” (reverse) sequence of the emPCR Amplification System (available at http://454.com/) to allow for hybridising to 454 GS-Junior DNA capture beads (454 Life Sciences - Roche Diagnostics, Basel, Switzerland). In the second set of treatments (Figure 1B), each species was individually PCR-amplified using three distinct Fusion Tags, and all PCR products were then pooled together by Fusion Tags either at equal, increasing or decreasing concentrations (Table 2B). In the third set of treatments (Figure 1C), the six environmental DNA samples artificially spiked with either none (control), one or five *A. amurensis* larvae were PCR-amplified using six specific Fusion Tags (see Table S3). All PCR amplifications were performed using Roche FastStart™ high fidelity taq and the thermocycling conditions detailed in the *Amplicon Library Preparation Method Manual* (Roche 2010; GS Junior Titanium Series).

### 454 Multiplex Analysis and Processing of Sequence Output

Fusion-tagged treatments 1–8 and 9–12 (Figure 1) were combined at an equimolar concentration of 25 ng/ul and processed using two separate pyrosequencing runs. These mixes underwent sequencing using Titanium chemistry (GS Rapid Library Prep, GS Junior Titanium emPCR Lib-L and GS Junior Titanium Sequencing Kits) on a GS Junior pyrosequencing system (Roche 454 Life Sciences, Branford, CT, USA), following manufacturer’s instructions.

The quality of the data obtained from the sequencing was verified using the Roche 454 Sequencing System Software version 2.7 (Roche 454 Life Sciences, Branford, CT, USA), including normalization, correction, and quality filtering steps to generate Standard Flowgram Format (SFF) files containing the base called read sequences and per-base quality scores as described in the Software manual.

The distribution of sequence lengths from all remaining sequences after quality check (SFF file format) was visualized using the Length Graph option in Geneious v5.5.6 [82]. The *SSU* sequences less than 425 bp and larger than 525 bp were deleted. All remaining sequences were oriented in the forward direction (5′-3′) and treatments were separated using the ‘Separate Reads by Barcode’ option in Geneious v5.5.6. Only sequences with perfect match on tags and a maximum of two errors on primers were included in the analysis. The amplified regions, excluding primers and tags, were retained for further analysis.

Identification of potential chimeric sequences was undertaken using the Chimera.Slayer sequence analysis pipeline available in the program Mothur v.1.26.0 [83]. For treatments one to six, Chimera.Slayer was run using the default settings, and chimeric sequences identified by comparing each treatment file to a reference sequence file containing a single *SSU* sequence per species. For treatments seven to twelve, chimeric sequences were identified using the ‘self’ option that uses the more abundant sequences in each treatment as a reference to which each query sequence is compared. Potential chimeric sequences were removed from further analysis.

All remaining sequences from treatments one through six were submitted to local BLASTn search in Geneious against the *SSU* reference sequence file, allowing a maximum hit of one and a BLAST e-value threshold of 10^−100^. Sequences from treatment seven through twelve were submitted to BLASTn in NCBI using the software BLAST2GO [84], allowing a maximum hit of 1 and BLAST e-value threshold of 10^−50^.

## Supporting Information

Figure S1 **Linear regression analysis.** Graphics show the correlations between number of sequences and relative abundances of starting DNA/PCR material obtained in **A**) decreasing DNA treatment 2, **B**) increasing DNA treatment 3, **C**) decreasing PCR treatment 5, and **D**) increasing PCR treatment 6.
(EPS)

Table S1 **Uncorrected genetic divergences of SSU sequences between MIS species.** Genetic divergences between the 12 species listed in Table 1 and based on the V1–V3 region of SSU. ^a^Numbers in brackets correspond to the species reference number shown in Figure 1.
(XLSX)

Table S2 **Detailed description of best BLASTn hits of all sequences obtained from environmental samples spiked with *Asterias amurensis* larvae.** Tables show the query sequences lengths, number of hits, minimum BLAST e-values, mean similarities, GenBank accession numbers, and the corresponding taxonomic description.
(XLSX)

Table S3 **List of fusion primers and fusion primer combinations used in this study.**
(XLSX)

DNA S1 **Reference DNA sequence alignment for the** ***COI*** **gene (fasta format).**
(FASTA)

DNA S2 **Reference DNA sequence alignment for the** ***SSU*** **gene (fasta format).**
(FAS)

DNA S3 **Pyrosequencing reads for treatments 1–6 (fasta format).**
(ZIP)

DNA S4 **Pyrosequencing reads for treatments 7–12 (fasta format).**
(ZIP)
